# A naturalistic database for understanding individual differences in affective responses to movie clips

**DOI:** 10.21203/rs.3.rs-10155655/v1

**Published:** 2026-08-05

**Authors:** Einat Liebenthal, Jeffrey M Girard, Manuel Jose Marte, Xuehu Wei, Colin Galvin, Yanmei Tie

**Affiliations:** Harvard Medical School; University of Kansas; Mclean Hospital; Harvard Medical School; Brigham and Women's Hospital; Harvard Medical School

## Abstract

Given the multidimensionality and dynamicity of movies, and open-ended nature of movie viewing, movie-based paradigms may hold potential for uncovering a richer array of human responses than possible with controlled experimentation. We created a database of movie clips, emotion ratings and derived features, and stimulus annotations, as a reference for studying normal and pathological emotional reactivity. We estimated the normative distribution of features derived from holistic ratings of negative and positive emotions, and real-time valence ratings (n = 108 healthy adults), of 22 short clips exhibiting unfolding social behaviors in diverse real-life situations. To capture dynamic and integrative aspects of emotional reactivity, we computed the intensity, complexity and intersubject synchronization of the real-time ratings, and intensity of the holistic ratings. We include biological (sex), individual (familiarity with the clips), and stimulus (gender representation in the clips) variables to facilitate investigation of their influence on the viewer’s emotional experience. We illustrate the potential of emotional reactivity profiling, and provide guidelines for stimulus selection for different applications, based on the real-time and holistic ratings.

## BACKGROUND & SUMMARY

Alterations in emotional reactivity are prevalent in mental illness. For example, blunted emotional reactivity to positive stimuli and events is a defining feature of anhedonia (loss of pleasure)^1^, and increased emotional reactivity to negative stimuli and events is an aspect of negative processing bias^2^, both hallmarks of depressive illnesses. However, it remains challenging to establish objective benchmarks against which to identify pathological alterations in emotional reactivity. This is largely due to the wide range and variability in normative emotional responses, which can be linked to biological factors (e.g., women are suggested to exhibit higher emotional reactivity than men^3, 4^), personality traits (e.g., neuroticism tends to be associated with higher negative reactivity and extroversion with higher positive reactivity^5^), cultural background^6^, and other individual factors (e.g., familiarity with the stimuli). Importantly, variability can arise not only in the intensity, but also the dynamic pattern of emotional reactivity, reflecting how readily and for how long one experiences a certain intensity of emotion.

Naturalistic paradigms employing stimuli and tasks that are less constraining and more similar to daily life experiences, such as viewing movie clips, are increasingly being used in neuroscientific research to evoke more naturalistic behaviors and neural responses. Given the multidimensional and dynamic structure of movie stimuli, and open-ended nature of movie viewing, movie-based paradigms may hold the potential to uncover a richer array of human responses than possible with traditional, controlled experimental paradigms using static unimodal stimuli (e.g., pictures). In this regard, movie-based approaches may be particularly well-suited for studying biological, trait, and pathological differences in multiple aspects of perception, cognition, and emotion. Indeed, neuroimaging studies using movie stimuli suggest that time-varying brain activation features tend to be more sensitive to biological differences^7^, and clinically-relevant alterations in emotional reactivity^8-10^, whereas stationary brain activation features have been shown to accurately predict trait-like phenotypes of cognition and emotion^11^. Thus, to capture the richness of the signal available in movie paradigms calls for characterization of not only stationary but also dynamic response properties, in relation to the dynamic and multidimensional movie properties. Nevertheless, few databases include moment-by-moment emotion ratings of movies, and those that do are limited by small participant samples (DECAF, COGNIMUSE^12, 13^), or report only the mean rather than full distribution of the ratings (TVNEWS^14^), precluding study of individual differences.

In the present work, we aimed to estimate the normative range and variability of both real-time and holistic emotional responses to a curated set of movie clips, as well as the effects of basic biological and individual variables and their interaction with relevant stimulus properties. The overarching objective was to create a database of clips, associated emotion ratings and derived features, and stimulus annotations, that could be used as a reference for studying normal and pathological phenotypes of emotional reactivity. We estimated the normative range and variability in emotional reactivity to 22 short clips (92 minutes in total duration) exhibiting unfolding social behaviors in diverse real-life situations, from live action films of different genres. Data were analyzed from 108 healthy adults (57 females, 51 males) who viewed the clips and rated in real-time the moment-by-moment intensity of the emotional valence, and at the end of the clip the intensity of specific emotions, that they felt. Previously, we published the real-time and holistic emotion ratings for this clip set in 83 participants^15^. Here, we extended the participant sample to balance it on sex. To capture dynamic aspects of emotional reactivity, we computed the distributions of not only the mean intensity, but also the complexity and intersubject synchronization of the real-time emotional valence ratings. We included additional individual (familiarity with the clips) and stimulus (representation of gender in the clips) variables to facilitate the investigation of their influence on the emotional experience of the viewer. To illustrate the use of the dataset for understanding individual differences in emotional reactivity to movie clips, we analyzed the effect of rater’s sex and give an example of emotional reactivity profiling for identifying phenotypes of negative affective bias. Finally, we provide guidelines for stimulus selection for different applications based on the real-time and holistic ratings.

We envision several uses for the database. The database can be used to model the effects of human factors (e.g., biological sex) and stimulus factors (e.g., visual, auditory, and linguistic movie features) on the intensity and dynamic pattern of the emotional responses. The database can serve as a normative reference for future studies wishing to use the movie clips and paradigm in different populations, e.g., to examine the effects on emotional reactivity of aging, mood disorder, personality traits, etc. The database can also support the interpretation of responses to the movie clips measured with other modalities, such as functional neuroimaging and electroencephalography to study the neurobiological basis of emotion. The accumulation of holistic and real-time multimodal responses to the same movie stimuli will serve to enhance our understanding of how individuals process and interpret stimuli and events under conditions that are more ecological than in traditional experimentation. We also envision that the database will facilitate and encourage the consideration of biological and individual variables in the design of future movie-based affective studies, such as balancing the stimulus set for the target population (i.e., including clips that appeal to the different demographics participating in the study). Given their richness and complexity, it’s not possible to fully characterize movie stimuli, or identify the full set of attributes that drive a movie response. However, characterizing the intensity and variance of responses they elicit along different dimensions, can capture the essence of movies without reducing their naturalistic quality. Thus, a more efficient set of stimuli can be selected for different applications based on movie response parameters, as we provide guidance here for affective studies.

## METHODS

### Participants

Participants were recruited from the community via the Rally with Mass General Brigham (MGB) online platform. Participants were 109 heathy adults reporting no neurological or psychiatric conditions, and no uncorrected vision or hearing deficits that could affect their ability to perform the paradigm. The sample consisted of 57 females and 51 males (one additional participant did not disclose sex or gender identity, and their data are not included in this report). The participants’ age ranged from 18 to 60 years (Mean (M) + Standard Deviation (SD) = 30.1 ± 11.3 years). The participants identified as White (52.7%), Asian (22.2%), Black/African American (16.7%), American Indian/Alaskan Native (1.9%), or Other (6.4%), and 12.9% identified as Hispanic. The female (F) and male (M) subgroups did not differ in age (F: 28.4 ± 9.7 years, M: 31.9 ± 12.9 years, t = 1.63, p = 0.11), or proportion of White versus non-White race classification (F: 52.6% White, M: 52.9% White, X^2^ = 0.001, p = 0.97).

### Study Procedures

Data collection was conducted remotely via Zoom video conferencing, as described in^15^. Participants viewed and rated up to 22 movie clips, in two sessions, each approximately 90 minutes long, with clip order randomized between participants. The number of participants rating each clip ranged from 94 to 104 (Median=100). For each clip, a one-sentence neutral description was first provided to orient participants to the scene they were about to see (all descriptions are given in the database). The participants then viewed the clip and rated in real-time, on a moment-by-moment basis (sampled at 30Hz), how it made them feel, on a single emotional valence scale ranging from −4 (very negative) to +4 (very positive), using the CARMA^16^ software. After viewing each clip, they answered three validation questions: (1) Were you able to hear the audio? (2) Was the picture clear? (3) Have you seen this clip before? They then provided holistic ratings of how the entire clip made them feel on an ordinal scale from 0 (‘very slightly or not at all’) to 4 (‘extremely’), in terms of the five positive affect items (alert, determined, enthusiastic, excited, inspired) and five negative affect items (afraid, distressed, nervous, upset, scared) of the Positive and Negative Affect Schedule – Short Form (PANAS-SF^17^). Finally, they answered three 4-alternative forced choice comprehension questions regarding the clip’s content.

### Movie Clip Stimuli

The stimuli consist of 22 clips from well-known, live-action feature films of different genres, that are freely available in movie libraries. The movie clips were selected based on the following criteria: 1) containing scenes with English spoken dialogue, 2) ostensibly spanning a range of valence and arousal levels and specific emotion categories, and 3) having demographically diverse actors (in terms of sex, age, and race) and varied content. The clips used in the study were extracted from a Blu-Ray copy to a separate MPEG-4 file with a frame width of 1920 pixels, frame rate of 23.976 fps, and audio sampling rate of 48 kHz. The clips were edited to 2-7 min long while maintaining the coherence of their narrative, and they were approximately equalized on perceived loudness. The English-language track was used without subtitles. The full list of clips with various descriptors is given in the ‘Stimuli_Descriptors’ file.

### Validation of emotion ratings

The ratings of clips for which participants reported that they couldn’t view or hear the clip properly were excluded (n=31 clips, or 1% of the data). The intensity of the emotion ratings was computed for the remainder of rater and clip combinations. The intensity of the holistic ratings was computed by averaging separately across the five positive and five negative affect items, to yield one positive and one negative holistic score. For the real-time ratings, the time series were resampled at 1Hz, the first 10 sec of response to each clip were dropped (as they were considered orientation time), and the remainder was averaged over time to yield a real-time valence intensity score. The inter-rater reliability of the real-time and holistic ratings was estimated as two-way intraclass correlation coefficients (ICCs) using the varde R package^18^. We used the average-measures consistency ICC for incomplete data or ICC(*Q,k*) which quantifies the reliability of the average of all available rater’s ratings, and consider values above .90 to be excellent.

### Emotional Reactivity Profiling and Outlier Detection

For constructing individual emotional reactivity profiles and identifying outliers, we computed several features. The standardized distance from the group mean (expressed in mean Z-scores) of the intensity of both the holistic and real-time ratings, was computed across all viewed movie clips for each rater. The complexity of the real-time ratings, varying from smooth (a flat line) to jagged, was calculated as the sample entropy, normalized using Robust Z-scores on the log-transformed values (to account for floor effects at zero). The intersubject correlation (ISC) of the real-time ratings was computed as the Pearson correlation (Fisher Z-transformed) between each participant’s time-series and the average time-series of all other participants (leave-one-out average). The raw ISC values are also provided, as they are meaningful without normalization. Low ISC values were considered to represent distraction and disengagement from movie-viewing.

Individuals who were outliers on the intensity of the holistic and real-time ratings, and complexity of the real-time ratings, were identified as those with Z-scores falling 2.5 standard deviations below or above the group mean. Outliers on synchrony of the real-time ratings were identified as those with intersubject correlation values below 0.3. A global Mahalanobis distance was also computed to identify outliers on combinations of these features.

### Normative Baselines

To establish a reference standard for future data collection with the same set of stimuli, we calculated the normative parameters (median, median absolute deviation - MAD) and the multivariate covariance structure of the emotional reactivity features in the current sample. Crucially, these norms were calculated using only the “valid” subset of participants, i.e., excluding those identified as outliers in terms of exceedingly low synchrony or low entropy of the real-time ratings, or global outliers. This ensured that the baseline represents “attentive human performance”, with minimal noise contamination.

### Stimulus features

For each clip, several features were computed to facilitate analyses of their influence on the emotion ratings, and facilitate the selection of balanced clip subsets for various applications. Because we observed sex effects on the emotion ratings (see Data Overview), we focused on stimulus features that relate to the representation of each sex in the clips and could potentially drive sex differences. The ratio of female and male raters reporting being familiar with the clip, was computed as a potential indicator of appeal and interest in each group. The ratio of female characters’ speaking time relative to the total (female + male) speaking time, and the ratio of female characters’ screen time relative to the total (female + male) screen time, were computed using the time stamps of the subtitles resampled at 2Hz. The effect of rater’s sex on the real-time valence ratings was estimated for each clip using generalized linear mixed models.

### Ethical statement

The study followed consenting procedures, and data collection and sharing protocols, mandated by the Mass General Brigham (MGB) Institutional Review Board (IRB). Participants provided informed consent to complete the study and have their deidentified data shared with other researchers under protocol 2022P000044. To further protect the privacy of the study participants and security of the data, we require users of the database to sign a data use agreement, as mandated under MGB IRB protocol 2023P001343.

## DATA RECORD

The dataset is organized as follows: 1) The ‘Ratings’ folder contains three files in .csv format, with the holistic and real-time emotion ratings for each participant and clip combination. The ‘holistic_by_item’ file contains the holistic emotion ratings for each of the 10 items of the PANAS-SF. The ‘holistic_by_scale’ file contains the ratings for the PANAS-SF positive and negative scales. The ‘valence_by_second’ file contains the second-by-second real-time valence ratings. 2) The ‘Valence_Summary’ folder contains three files: The ‘features_per_rater’ .csv file contains features derived from the real-time ratings for each participant across clips (in standardized z-scores), including the mean intensity and standard deviation, the entropy, and intersubject correlation. This file also contains for each participant and clip, their familiarity with the clip (yes/no response) and accuracy score on the three comprehension questions. The ‘features_per_combo’ file contains the raw scores for these variables for each participant and clip. The ‘Valence_by_Sex’ .docx file contains plots of the momentary mean and 95% confidence intervals of the real-time emotional valence ratings for each clip stratified by sex, and examples of four scenes that elicited sex differences. 3) The ‘Demographics’ folder contains a .csv file with the sex, race, ethnicity, and age for each participant. 4) The ‘Stimuli’ folder contains a ‘Video’ subfolder with the clips used in the study in .mp4 format, and a ‘Subtitles’ folder with the clip subtitles in .srt format. The ‘Stimuli_Descriptors’ .docx file contains two tables. Table SD1 lists for each clip, the title of the originating movie, director, genre and production year, ratio of female speaking time, ratio of female screen time, ratio of female participants familiar with the movie, and size of sex effect on real-time ratings. Table SD2 lists for each clip, their suitability for studying negative affective bias, based on the mean and standard deviation of the holistic negative ratings, and reliability of the real-time emotional valence ratings. DynAMoS: The Dynamic Affective Movie clip Database for Subjectivity Analysis is hosted at dynamos.mgb.org. The database is available to academic researchers for non-profit use, upon request via a link on the website.

## DATA OVERVIEW

**Figure 1:**
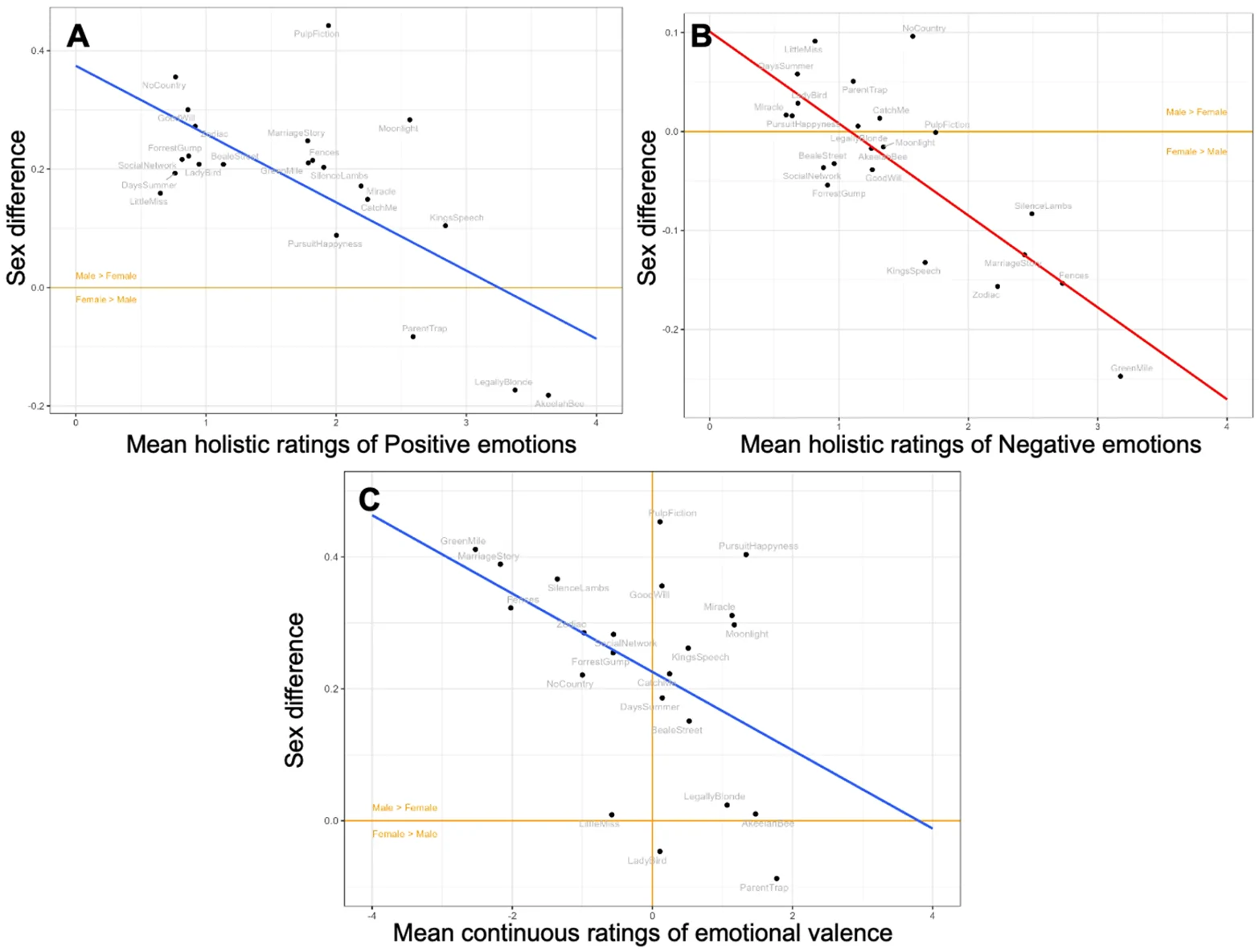
Effect of sex on the holistic ratings of (**A**) positive and (**B**) negative emotions, and (**C**) the real-time ratings of emotional valence. In each plot, the x-axis represents the mean ratings across all participants. The y-axis represents the sex difference in ratings, with positive values indicating a higher mean in males relative to females, and negative values indicating a higher mean in females relative to males. Clips for which the mean difference in ratings between males and females was significant in independent models are denoted with *(p<0.05) or ** (p<0.01). The linear fit lines in each plot illustrate the relationship between the overall valence of each clip and the sex differences it elicited.

**Figure 2:**
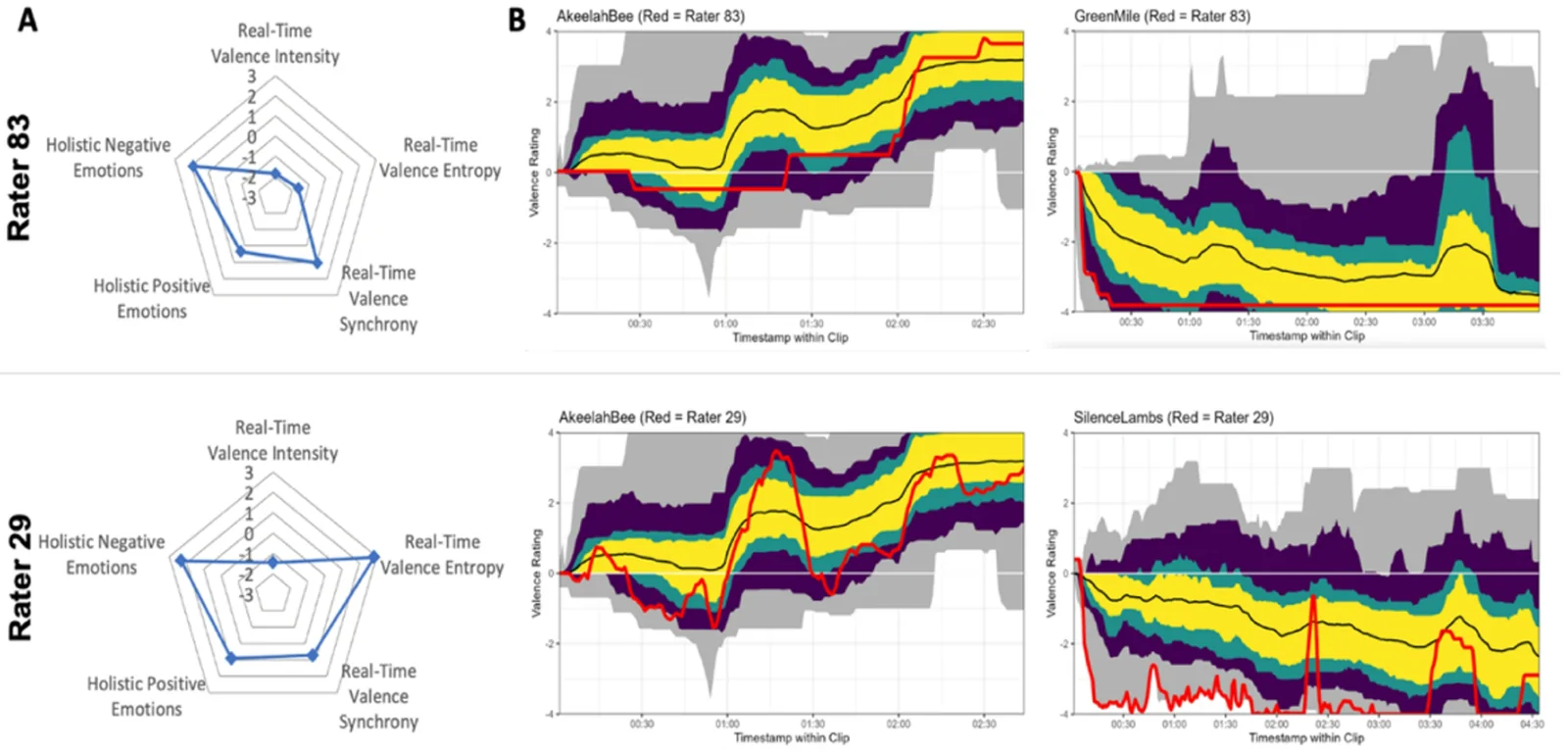
Emotional reactivity profiles of two raters falling in the top 10% most negative in terms of the intensity of the real-time and holistic ratings, but differing widely in terms of the complexity of the real-time ratings. **(A)** Radar plots showing for each rater, the standardized distance from the group mean (in Z-scores) of the intensity, entropy and synchrony of the real-time valence ratings, and of the intensity of the holistic negative and positive emotion ratings. **(B)** Chromodoris plots showing the momentary real-time valence ratings for each rater (red trace), overlaid on the group mean (black trace) and 50, 70, 90 and 100% of the distribution, for one clip with overall positive valence (from “Akeelah and the Bee”, left), and one clip with overall negative valence (from “Silence of the Lambs” and “The Green Mile”, right).

To illustrate the independent value of the real-time ratings for understanding individual differences in emotional reactivity, we analyzed the effect of rater’s sex and give an example of two individual profiles resembling in their negative tendency but differing widely in the temporal pattern of emotional responses. Generalized linear mixed models of sex effects on the holistic ratings (across clips) revealed a trend toward higher positive emotions in males than females (coefficient=0.17, 95% CI=−0.02, 0.37, z=1.75, p=0.08), and no effect on the negative emotions (coefficient=−0.03, 95% CI=−0.21, 0.14, z=0.36, p=0.72). The real-time valence ratings (across all clips and time points) were significantly more positive in males than females (coefficient=0.23, 95% CI= 0.05, 0.42, z=2.45, p=0.01). Fitting a linear line to the sex difference data revealed an interaction with the overall valence of each clip: clips rated more negatively (e.g., ‘Green Mile’) tended to elicit relatively more negative ratings in females than males, whereas clips rated more positively (e.g., ‘Parent Trap’) tended to elicit relatively more positive ratings in females than males (Fig. 1). This pattern is consistent with generally greater real-time emotional reactivity in females relative to males. Furthermore, sex differences in the real-time ratings were not sustained, rather occurred in specific moments of specific clips (see Figs. VS1 and VS2 in Valence_by_Sex.docx). Example profiles of two raters falling in the top 10% most negative in terms of the intensity of both the real-time and holistic ratings, but differing widely in terms of the complexity of the real-time ratings, are shown in Fig. 2. Rater 83 fell in the bottom 10% in terms of real-time complexity, reflecting a sustained, mostly negatively biased, rating pattern. Whereas rater 29 was an outlier with exceedingly high real-time complexity, reflecting a pattern of frequent switching between extreme negative and positive ratings, in relation with fluctuations in the emotional salience of the stimuli. Extremely low complexity of response could potentially reflect low motivation and diminished interest (as in anhedonia), whereas high complexity of response could reflect greater volatility and impulsiveness, or hypervigilance. Overall, the implications are that time-resolved methods and dynamic features provide substantive complementary information for characterizing emotional reactivity. Furthermore, there is a need for developing sex-specific norms and stimulus materials for both basic and translational applications in affective science.

## TECHNICAL VALIDATION

The response accuracy to the comprehension questions was high across subjects and clips (95.7%±5.2%), suggesting that overall understanding of the gist of each clip’s plot was good.

### Quality of emotion ratings

**Figure 3:**
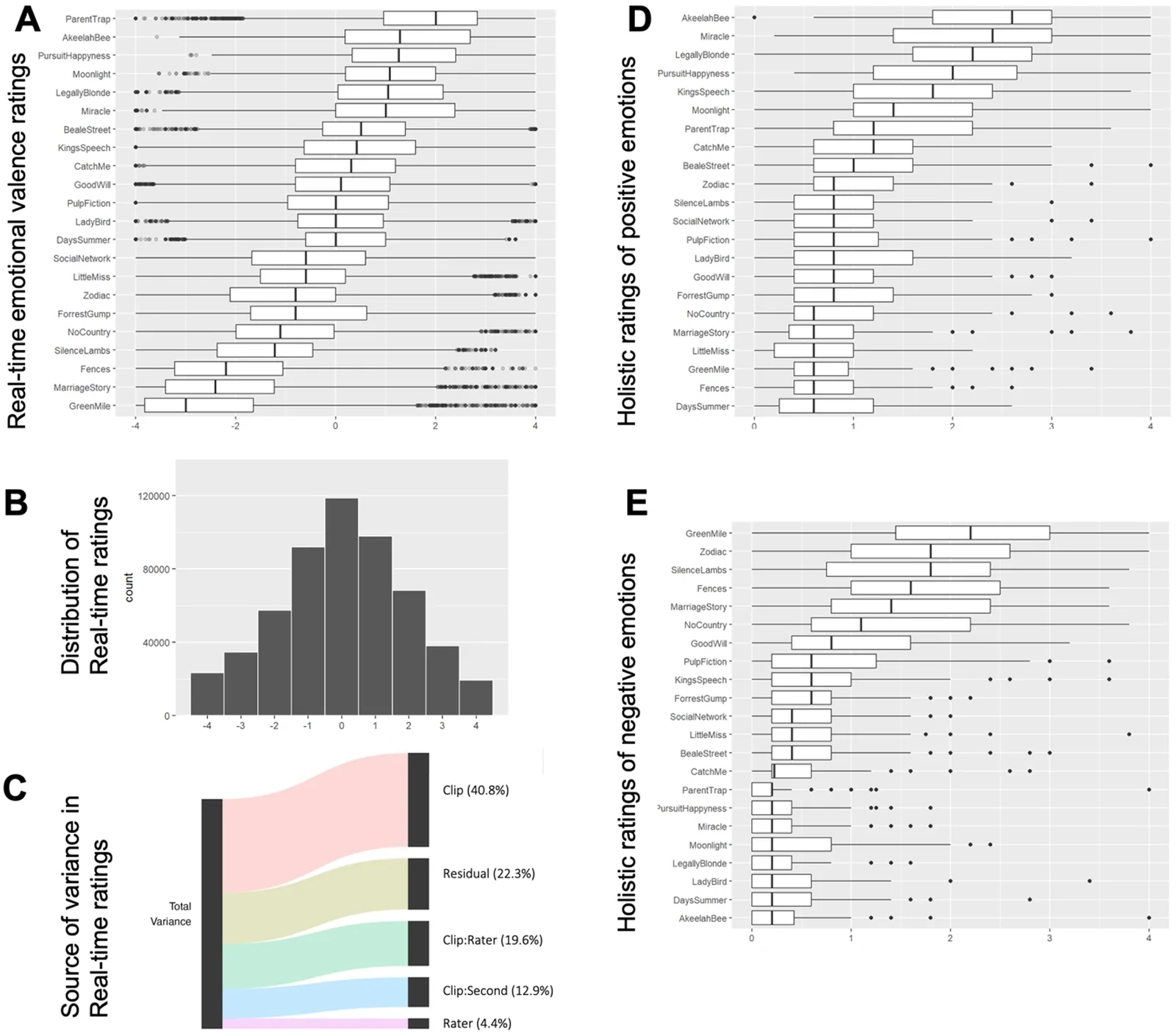
**(A)** Distribution across participants of the real-time emotional valence ratings for each clip, and (**B**) across clips. The emotional valence was rated in real-time while watching each clip, on a continuous scale from −4 (very negative) to +4 (very positive). (**C**) Sources of variance in the real-time ratings. (**D**) Distribution across participants of the mean scale scores on the five Positive Affect items (alert, determined, enthusiastic, excited, inspired), and (**E**) the five Negative Affect items (afraid, distressed, nervous, scared, upset), each rated holistically on an ordinal scale from 0 to 4 after viewing each clip.

The clips in the database elicited a wide range of emotional responses that were overall well balanced in terms of positive, neutral, and negative content. The interrater reliability was high for both the holistic and real-time ratings. For the real-time ratings, the intersubject synchrony was high and the entropy was low (**Table 1**), indicating an overall reliable and predictable dynamic pattern of response between subjects across clips. Specifically, the distribution of the holistic and real-time ratings confirmed that the clips elicited a wide range of dynamic emotional valence ratings, and specific positive and negative emotions (Fig. 3), as designed. The holistic ratings of positive emotions varied from 0.82±0.67 for ‘Days of Summer’ to 2.43±0.84 for ‘Akeelah’, and the holistic ratings of negative emotions varied from 0.37±0.51 for ‘Akeelah’ to 2.19±1.0 for ‘Green Mile’ (on a discrete scale of 0-4). The momentary means of the emotional valence rating varied from −2.53±1.51 for ‘Green Mile’ to 1.82±1.39 for ‘Parent Trap’ (on a continuous scale of −4 to +4). The inter-rater reliability, estimated as the balanced average-measures consistency intraclass correlation (ICC)^19^, was very high for the holistic ratings of positive (ICC=0.992, 95% CI=[0.984, 0.996]) and negative (ICC=0.989, 95% CI=[0.979, 0994]) emotions across clips. The inter-rater reliability of the moment-by-moment emotional valence ratings was also high (ICC > 0.940) for all clips except one (Little Miss’, ICC=0.89, 95% CI=[0.853, 0.913]). Across all clips, the dynamic ratings were normally distributed, with low skewness and kurtosis (Fig. 3B). Variance decomposition indicated that the largest sources of variance in the dynamic valence ratings were differences between the movie clips (40.8%) and clip segments (12.9%), while differences between raters contributed only 4.4% of the variance. However, the interaction of clip by rater contributed 19.6% of the variance, suggesting that the extent of individual variance in the ratings was greater for certain clips than others (Fig. 3E).

### Normative baselines

Six raters were identified as outliers (≥2.5 standard deviations from the mean) on one of the emotion rating features, and two were global outliers (deviating on a combination of the features). Two other raters showed very low real-time synchrony (<0.3), suggesting that their ratings may be unreliable.

The parameters (median, median absolute deviation) and multivariate covariance structure of the real-time and holistic responses in the current sample of healthy adults (excluding outliers) across all clips, are provided as a normative reference for future work (**Table 1**). For the real-time ratings, the intersubject synchrony was high (0.722±0.126), indicating an overall reliable dynamic pattern of response between subjects across clips. The entropy was centered around negative values (−2.419±0.497), suggesting an overall less complex or more predictable trajectory of ratings over the course of each clip. The valence was overall centered around neutral (−0.025±0.437), suggesting a good balance in the intensity of movie scenes judged as negative, positive, and neutral across clips. For the holistic ratings, the intensity was slightly higher for positive (1.181±0.416) than negative (0.771±0.421) emotions.

Pearson correlation between the features revealed a stronger relationship between the intensity of the positive and negative holistic ratings (r=0.48) than between the intensity of the real-time and holistic positive (r=0.35) and negative (r=−0.29) ratings. The relationship between the intensity and complexity (r=0.26) of the real-time ratings was weaker (**Table 1**). The pattern of correlation between features suggests that the holistic and real-time ratings capture different aspects of emotional reactivity, with both the intensity and complexity of the real-time ratings contributing distinct characteristics.

## Supplementary Material

This is a list of supplementary files associated with this preprint. Click to download.

- outlierexploration2026.html
- Table1.docx

## Data Availability

The database, including the deidentified raw emotion ratings, derived features, stimuli, and associated metadata such as participant demographic information, as described in the data record, will be available for sharing with academic researchers for non-profit use, upon request via a public link and signing of a data use agreement (DUA). The DUA form can be found in the ‘Request Access’ tab on the dynamos.mgb.org website.

## CODE AVAILABILITY

The analysis code, including R scripts for data processing and feature generation, are provided as supplementary materials.
